# Expanding the applications of immune checkpoint inhibitors in advanced lung cancer beyond disease progression

**DOI:** 10.3389/fimmu.2023.1266992

**Published:** 2023-09-15

**Authors:** Chao Chen, Xi Xiong, Ying Cheng, Haiyun Gen, Wenqiang Zhu, Fei Zhang, Chuandong Zhu, Siqi Han, Xiufeng Liu

**Affiliations:** ^1^ Department of Oncology, Jinling Hospital, Nanjing Medical University, Nanjing, China; ^2^ Department of Hepatology, Jinling Hospital, Nanjing Medical University, Nanjing, China; ^3^ Department of Oncology, Jiangsu Province Hospital of Chinese Medicine, The Affiliated Hospital of Nanjing University of Chinese Medicine, Nanjing, China; ^4^ Department of Surgical Oncology, Jiangsu Province Hospital of Chinese Medicine, The Affiliated Hospital of Nanjing University of Chinese Medicine, Nanjing, China; ^5^ Department of Clinical Laboratory, Jinling Hospital, Nanjing Medical University, Nanjing, China; ^6^ Department of Oncology, the Second Hospital of Nanjing, Nanjing University of Chinese Medicine, Nanjing, China

**Keywords:** advanced lung cancer, PD-1, treatment beyond progression, immunotherapy, retrospective study

## Abstract

**Background:**

Immunotherapy, particularly the utilization of immune checkpoint inhibitors (ICIs), assumes a pivotal role in the comprehensive management of advanced lung cancer. There has been substantial deliberation regarding the appropriateness of extending ICIs treatment beyond the point of disease progression. This study delves into the potential benefits of sustained utilization of ICIs subsequent to disease progression in patients.

**Methods:**

A retrospective analysis was conducted on a cohort of 248 patients diagnosed with advanced lung cancer who received treatment with ICIs. The study population comprised 99 patients in the treatment beyond progression (TBP) group and 42 patients in the non-treatment beyond progression (NTBP) group. Parameters including progression-free survival (PFS), overall survival (OS), objective response rate (ORR), and disease control rate (DCR) were assessed. The Cox proportional hazard regression model was employed to analyze prognostic factors related to immunotherapy.

**Results:**

Patients undergoing primary treatment with PD-1/PD-L1 inhibitors exhibited a median progression-free survival (mPFS) of 5.3 months. In the context of disease progression, a comparison between the TBP and NTBP groups was performed with respect to mPFS. The results demonstrated that the TBP group manifested an mPFS of 8.6 months, contrasting with the NTBP group’s mPFS of 4.0 months (p=0.028). The mean overall survival (mOS) in the TBP group exhibited a statistically significant increase in comparison to the NTBP group (14.1 months vs. 6.0 months, p=0.028). Evaluation of the objective response rate (ORR) between the TBP and NTBP groups revealed a substantial distinction. The TBP group displayed an ORR of 12.1%, while the NTBP group exhibited a lower ORR of 2.4%. The statistical analysis yielded a p-value of 0.068, signifying a notable trend towards significance. The disease control rate (DCR) was also assessed and exhibited a noteworthy variance between the two groups, with a higher DCR of 92.9% in contrast to 71.4% in the control group (p = 0.001).

**Conclusion:**

Subsequent to ICIs treatment, a subset of patients may derive continued benefits from anticancer therapy, notwithstanding the progression of their advanced lung cancer.

## Introduction

Lung cancer stands as the leading cause of tumor-related mortality on a global scale. The notable decrease in lung cancer mortality observed in recent years can be largely attributed to substantial advancements in early detection, molecular targeted therapy, and immunotherapy. Nevertheless, the 5-year survival rate for patients with non-small cell lung cancer (NSCLC) remains modest, hovering below 20% (1). Among the remarkable breakthroughs in lung cancer treatment, immunotherapy has emerged as a pivotal development. PD-1, a type I transmembrane protein comprising 268 amino acids from the immunoglobulin B7CD28 family, plays a crucial role. PD-1, with its primary ligand PDL1 expressed widely in antigen-presenting and non-blood cells, operates as a negative regulator in human immune responses. Its distinct therapeutic mechanism sets it apart from conventional chemotherapy and radiotherapy, activating the immune system to combat cancer cells. Notably, immune checkpoint inhibitors, including PD-1 and PD-L1 inhibitors, exemplify immunotherapy’s forefront, stimulating the immune system through T cell activation against tumors (2, 3).

The PD-1/PD-L1 signaling pathway exerts significant influence on T cell activation and exerts control over the generation of growth factors and cellular proliferation (4). Pathological stimulation of this pathway impedes T lymphocyte activation and replication, fosters regulatory T lymphocyte development, and facilitates evasive tactics by neoplastic cells against immune recognition and elimination (5). The utilization of immune checkpoint inhibitors (ICIs) has ushered in a new era of immunotherapy for advanced NSCLC in clinical settings (6).

Distinguished from conventional chemotherapy and targeted therapy, the anti-neoplastic mechanism of immune checkpoint inhibitors (ICIs) necessitates distinct approaches to evaluating treatment effectiveness. Due to the potential for atypical delayed responses and pseudo-progression in tumor patients undergoing ICIs treatment, assessment of progression-free survival (PFS) and objective response rate (ORR) for solid tumors relies on the Response Evaluation Criteria in Solid Tumors (RECIST) v1.1. Nevertheless, the assessment of immunotherapy presents challenges, as immune-related Response Evaluation Criteria in Solid Tumors (irRECIST) and modified immune-related Response Evaluation Criteria in Solid Tumors (iRECIST) remain infrequently employed in clinical practice (7, 8).

Traditionally, disease progression in the context of immunotherapy signifies treatment failure and often leads to its discontinuation. However, reports from the literature suggest that even in metastatic renal cell carcinoma cases, patients may continue receiving PD-1 monoclonal antibodies post-disease progression, potentially yielding survival benefits (9).

Amidst the backdrop of ongoing debates within various studies, a pertinent discussion surrounds the potential advantages of maintaining immunotherapy with immune checkpoint inhibitors (ICIs) for patients with advanced lung cancer following disease progression. The relevance of continuing immunotherapy after disease progression diminishes in patients with advanced lung carcinoma who have previously undergone immunotherapy or a combination of immunotherapy and chemotherapy, and reports vary in their findings (9–13). Our study endeavors to validate the clinical efficacy of ICIs in lung cancer patients facing disease progression subsequent to ICIs therapy.

## Patients and methods

### Patients

A retrospective analysis was conducted on a cohort of 248 patients with advanced lung cancer who underwent treatment with immune checkpoint inhibitors (ICIs) at Nanjing Jinling Hospital between January 1, 2018, and October 31, 2022. Inclusion criteria were as follows: a) age ranging from 18 to 80 years; b) confirmed pathological diagnosis of small cell lung cancer or non-small cell lung cancer, including adenocarcinoma, squamous cell carcinoma, adenosquamous cell carcinoma, etc.; c) accordance with the eighth edition of the cancer TNM classification, with patients at stage IIIB or IV or those with recurrent disease; d) receipt of at least 2 cycles of ICIs treatment with a minimum interval; e) absence of intolerable toxic reactions from prior immunotherapy; f) Eastern Cooperative Oncology Group (ECOG) score less than 3; g) receipt of PD-1 monoclonal antibodies at least twice following disease progression. The final follow-up date was April 01, 2023. All participants provided informed consent, adhered to the ethical principles outlined in the Helsinki Declaration, and received approval from the hospital’s ethics committee.

### Study design

Oncologists and radiologists collaborated to perform imaging evaluations, classifying efficacy into complete remission (CR), partial remission (PR), disease stability (SD), and disease progression (PD) based on RECISTv1.1 criteria. The objective response rate (ORR) was calculated by combining complete remission (CR) and partial remission (PR), while the disease control rate (DCR) was computed using the sum of complete remission, partial remission, and standard deviation (CR+PR+SD).

The initial assessment of disease response occurred after the completion of two treatment cycles or earlier if clinically indicated. ORR analysis was based on the best overall response (BOR), encompassing both partial and complete response rates. Disease control rate (DCR) was determined by evaluating the rates of partial remission, complete remission, and disease stability.

The PFS was defined as the time between commencement of immune checkpoint inhibitor treatment and disease progression. PFS2 represented the interval between the first occurrence of immunotherapy-induced progression and the subsequent progression or mortality from any cause. Overall survival (OS) was calculated from the first progression after ICIs treatment to mortality from any cause. Baseline characteristics evaluated included age, gender, smoking history, surgery, radiotherapy, ECOG status, histology, brain metastasis, bone metastasis, and liver metastasis.

Safety evaluations encompassed all eligible patients, with adverse events (AEs) graded according to the National Cancer Institute Common Terminology Criteria for Adverse Events, version 5.0.

### Statistical analysis

The statistical analysis of the data in this research was carried out utilizing SPSS 22.0 (Chicago, IL, USA) or GraphPad Prism 8.0 (San Diego, California, USA). To assess differences in continuous variables between the treatment and control groups, the Mann-Whitney U test was employed. For discrete data group comparisons, the Pearson χ² test or Fisher’s exact test was applied. PFS and OS rates were calculated using the Kaplan-Meier method, with disparities between groups evaluated using the log-rank test. The Cox proportional hazard regression model was used for both univariate and multivariate analyses of prognostic factors associated with overall survival post-immunotherapy. Relative risk between groups was evaluated through the hazard ratio (HR) and a 95% confidence interval (CI). A statistical significance level of α = 0.05 was set for all conducted tests.

## Results

### Patient clinical characteristics

**Figure 1:**
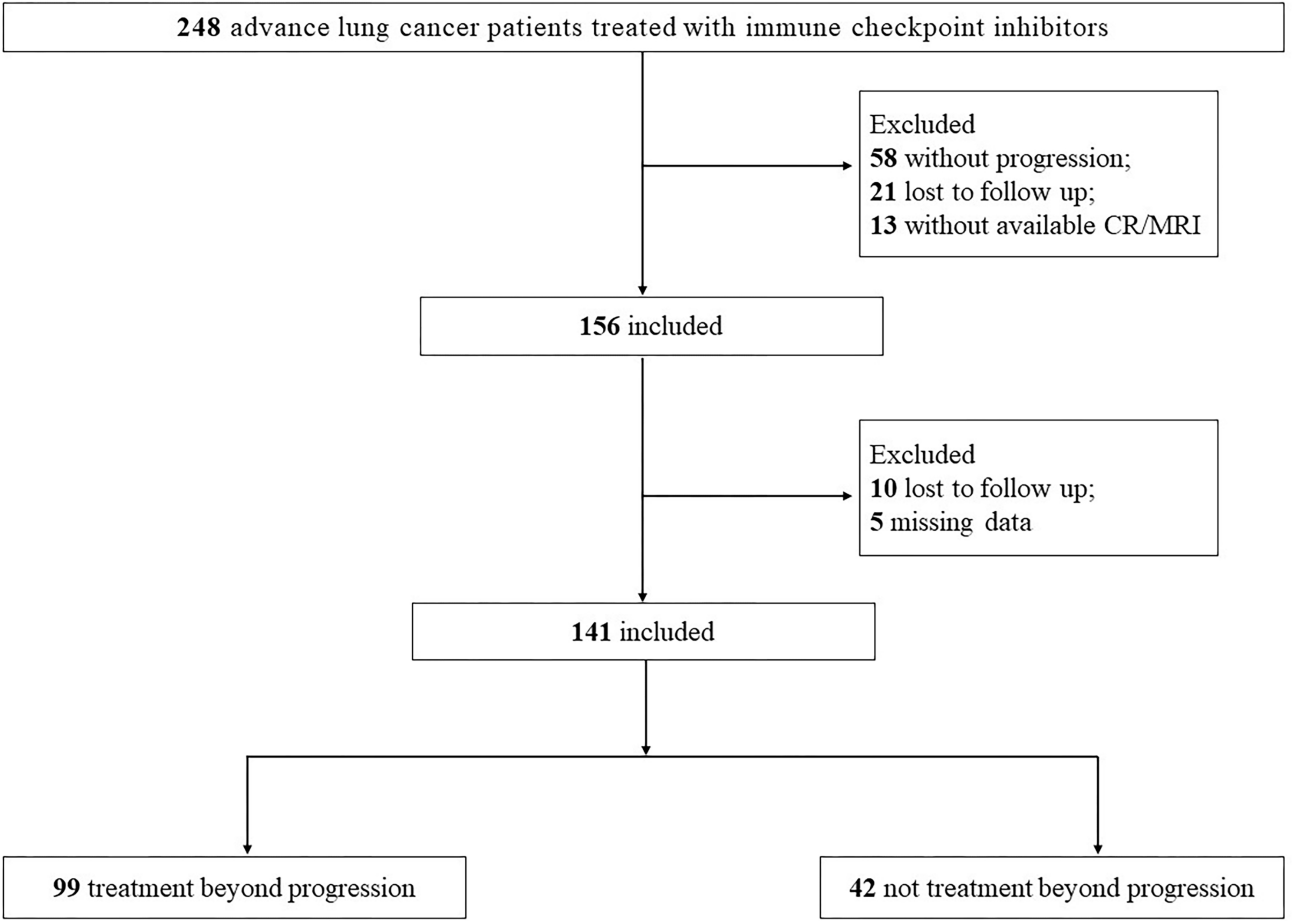
Flow chart of the study.

The patient selection process is depicted in **Figure 1**. Between January 1st, 2018, and October 31st, 2022, a total of 248 patients with advanced lung cancer received ICIs treatment at Nanjing Jinling Hospital. Following RECIST 1.1 criteria, disease progression was observed in 156 patients, while 10 patients were lost to follow-up, and 5 patients had incomplete data. Ultimately, the analysis encompassed 141 patients, comprising 99 patients in the Treatment beyond Progression (TBP) group, which is refers to the practice of continuing a treatment regimen without any interruption or cessation, even after disease progression has been observed. This approach involves maintaining the therapy without a predefined stop time, allowing for ongoing management and potential benefits for the patient and 42 patients in the Non-Treatment beyond Progression (NTBP) group.

The characteristics of the patients are summarized in **Table 1**. The TBP group exhibited an average age of 62.9 ± 9.5 years, with 16 female patients (16.2%), 35 patients (35.4%) having undergone surgical treatment, and 47 patients (47.5%) receiving radiotherapy. In contrast, the NTBP group had an average age of 63.0 ± 9.7 years, comprising 11 female patients (26.2%), 11 patients (26.2%) with a history of surgical treatment, and 21 patients (50.0%) who had received radiotherapy.

**Table 1:** Clinical information of included patients.

Factors	NTBP,n=42(%)	TBP,n=99(%)	P value
Age			0.929
	63.0 ± 9.7	62.9 ± 9.5	
Gender			0.166
Female	11(26.2)	16(16.2)	
Male	31(73.8)	83(83.8)	
Smoking history			0.722
Current/Former	14(33.3)	30(30.3)	
Never	28(66.7)	69(69.7)	
ECOG PS			0.011
0	11(26.2)	38(38.4)	
1	23(54.8)	57(57.6)	
2	8(19.0)	4(4.0)	
Surgery			0.179
No	32(76.2)	64(64.6)	
Yes	10(23.8)	35(35.4)	
Radiotherapy			0.784
No	21(50.0)	52(52.5)	
Yes	21(50.0)	47(47.5)	
Pathology			0.084
Small Cell	6(14.3)	16(16.2)	
Squamous	13(31.0)	28(28.3)	
Adenocarcinoma	19(45.2)	54(54.5)	
Other	4(9.5)	1(1.0)	
PD-1 inhibitors			0.914
Atezolizumab	1(2.4)	3(3.0)	
Durvalumab	4(9.5)	4(4.0)	
Camrelizumab	19(45.2)	49(49.5)	
Nivolumab	2(4.8)	6(6.1)	
Pembrolizumab	3(7.1)	4(4.0)	
Toripalimab	3(7.1)	10(10.1)	
Tislelizumab	6(14.3)	14(14.1)	
Sintilimab	4(9.5)	9(9.1)	

### Clinical outcomes

**Figure 2:**
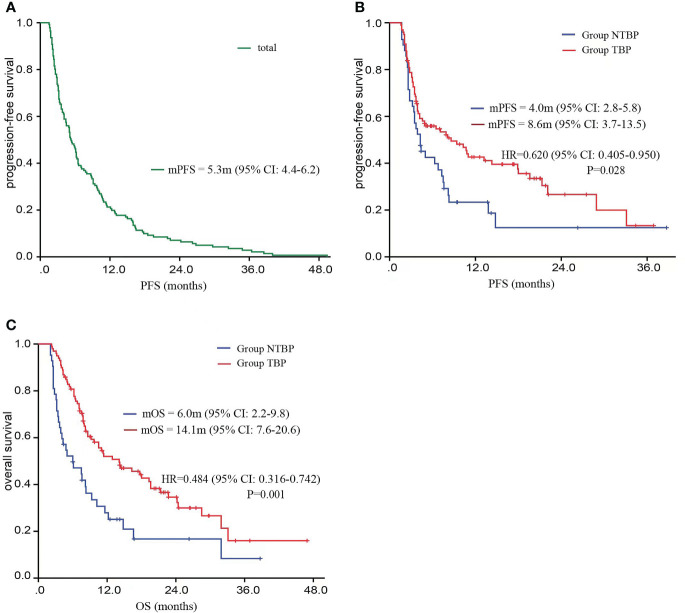
Kaplan–Meier curves of PFS and OS in advance lung cancer patients according to response to ICIs. **(A)** the PFS of advance lung cancer patients received ICIs treatment at first time. **(B)** the PFS of advance lung cancer patients in TBP and NTBP. **(C)** the OS of advance lung cancer patients in TBP and NTBP.

**Figure 3:**
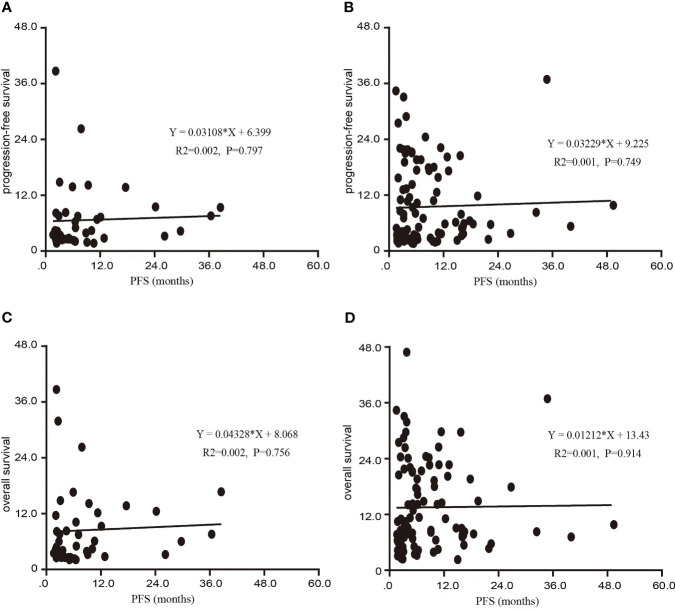
Scatter diagram of the association between PFS1 and PFS 2, OS in this study. **(A)** Scatter diagram of the association between PFS1 and PFS in NTBP. **(B)** Scatter diagram of the association between PFS1 and PFS in TBP. **(C)** Scatter diagram of the association between PFS1 and OS in NTBP. **(D)** Scatter diagram of the association between PFS1 and OS in TBP.

Among the 141 patients included in this clinical trial, the mPFS after initial treatment with PD-1/PD-L1 therapy was 5.3 months (95% CI: 4.4-6.2). Subsequent to disease progression, the TBP group exhibited notably extended mPFS in comparison to the NTBP group (8.6 vs. 4.0 months, HR=0.620, 95% CI: 0.405-0.950, P=0.028). Furthermore, the TBP group demonstrated a significantly higher mOS in contrast to the NTBP group (14.1 vs. 6.0 months, HR=0.484, 95% CI: 0.405-0.950, P=0.028), as depicted in **Figure 2**. Notably, no significant correlation emerged between PFS2 and PFS1 in the NTBP group (Y = 0.03108*X + 6.399, R²=0.002, P=0.797). Similarly, no significant correlation was observed between mOS and PFS1 (Y=0.04328*X+8.068, R²=0.002, P=0.756). While no statistically significant correlation emerged between PFS2 (Y = 0.03229*X + 9.225, R²=0.001, P=0.749) and OS (Y = 0.01212*X+13.43, R²=0.001, P=0.914) with PFS1 in the TBP group, a consistent trend was evident (**Figure 3**).

**Figure 4:**
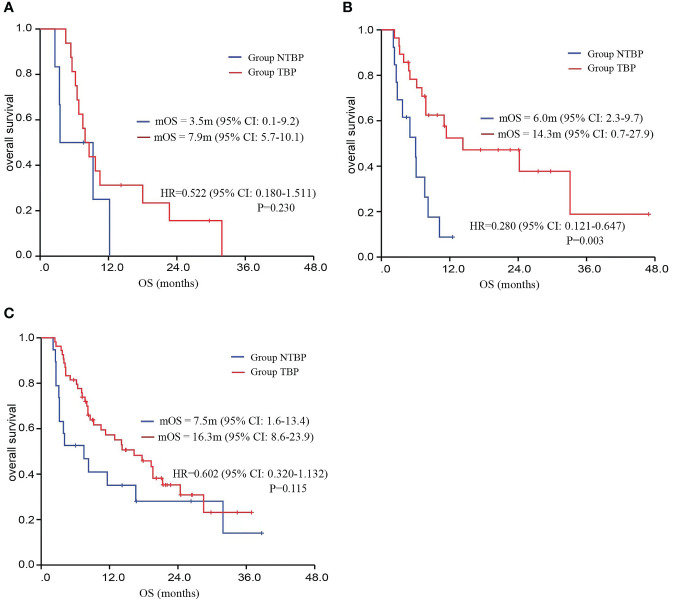
Kaplan–Meier curves for OS comparing TBP and NTBP in different pathological types of lung cancer patients. **(A)** Kaplan–Meier curves of OS comparing TBP and NTBP in small cell lung cancer. **(B)** Kaplan–Meier curves of OS comparing TBP and NTBP in squamous cell lung carcinoma. **(C)** Kaplan–Meier curves of OS comparing TBP and NTBP in lung adenocarcinoma.

**Figure 5:**
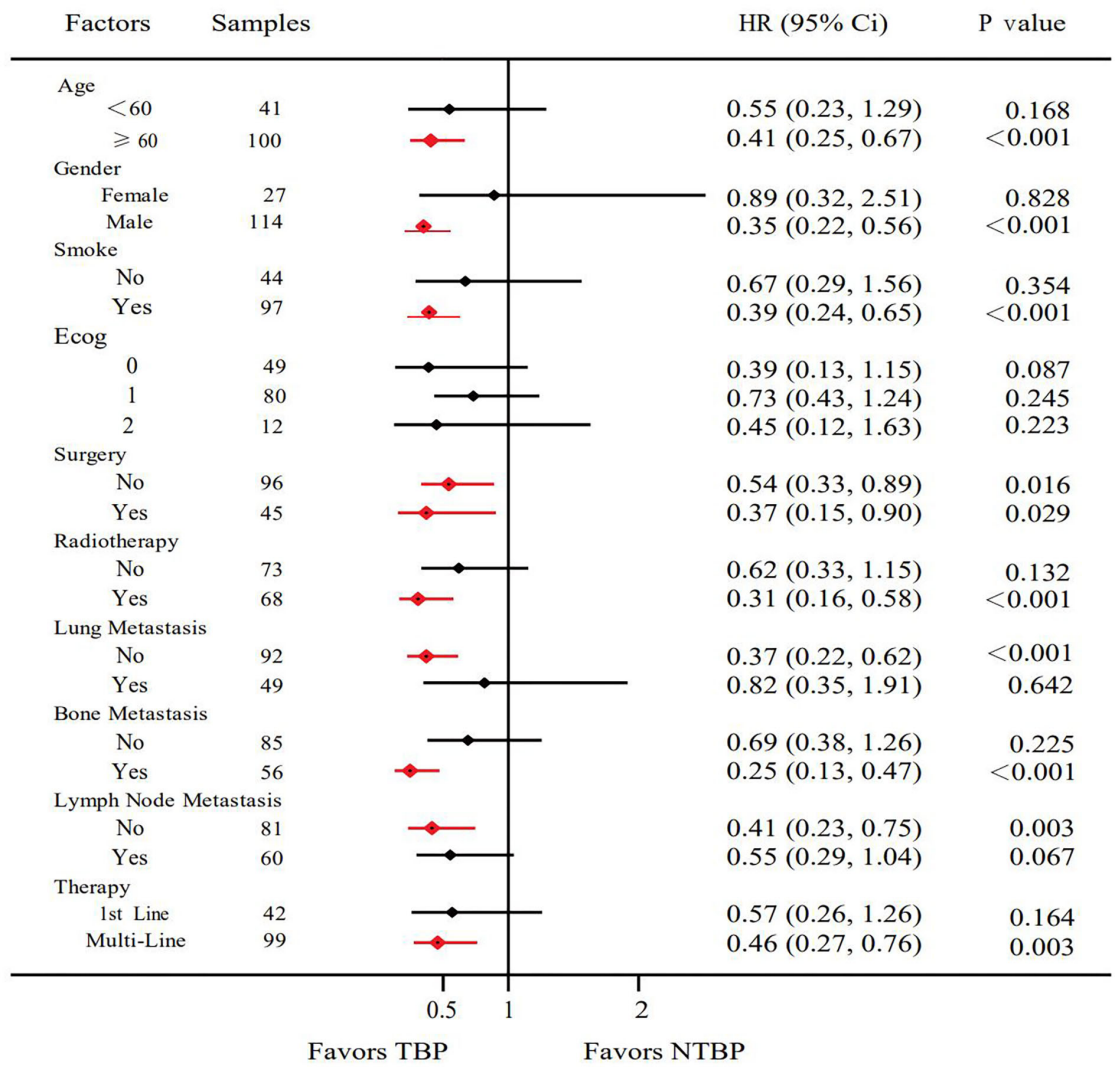
Forest plots for the clinical characteristics in TBP and NTBP.

Based on pathological classification, small cell lung cancer patients in the TBP group (16 cases) displayed a lengthier mOS trend compared to the NTBP group (6 cases) (7.9 vs. 3.5 months, HR=0.522, 95% CI: 0.180-1.511, P=0.230). In the cohort diagnosed with squamous cell carcinoma and treated with TBP (28 cases), a prolonged mOS was evident relative to NTBP (13 cases) (14.3 vs. 6.0 months, HR=0.280, 95% CI: 0.121-0.647, P=0.003). Similarly, patients diagnosed with adenocarcinoma and treated with TBP (54 cases) exhibited a higher mOS than the NTBP group (19 cases) (16.3 vs. 7.5 months, HR=0.602, 95% CI: 0.320-1.132, P=0.115), though statistical significance was not reached (**Figure 4**). The TBP group displayed an ORR of 12.1% compared to 2.4% in the NTBP group, yielding a p-value of 0.068. Furthermore, the disease control rate (DCR) was 92.9% versus 71.4% (p-value = 0.001), as outlined in **Table 2**. Analysis of the forest plot involving patient age, gender, smoking status, ECOG performance status, therapy line, radiotherapy, presence of lung metastasis, bone metastasis, and lymph node metastasis indicated the TBP group’s enhanced efficacy (**Figure 5**).

**Table 2:** Summary of efficacy of PD-1Treat beyond progression.

	NTBP,n=42	TBP,n=99	P value
Objective response rate, N (%)	1(2.4)	12(12.1)	0.068
Disease control rate, N (%)	30(71.4)	92(92.9)	0.001
Best overall response, N (%)			0.003
Progressive disease	12(28.6)	7(7.1)	
Stable disease	29(69.0)	80(80.8)	
Partial response	1(2.4)	12(12.1)	
Complete response	0	0	

TBP, treatment beyond progression; NTBP, not treatment beyond progression.

### Safety

**Figure 6:**
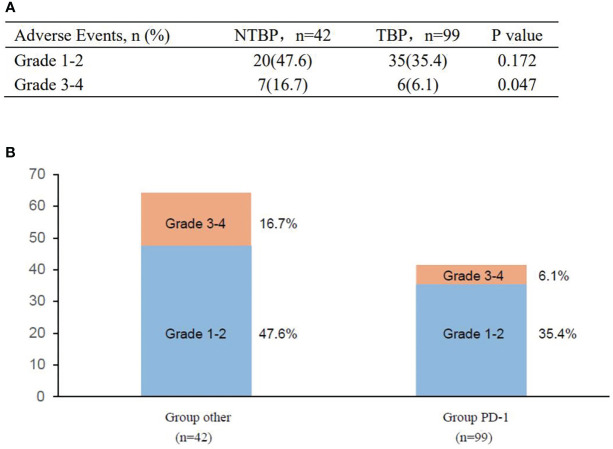
Adverse events in the study before and after PD. **(A)** Adverse events of special interest. **(B)** Treatment-related adverse events.

A comprehensive safety assessment was conducted on all patients (**Figure 6**). The incidence of immune-related adverse events in the TBP group paralleled that of the PRE-PD group. Primary severe adverse effects encompassed immune-mediated pneumonia, dermatitis, and asthenia. The TBP group did not manifest an elevation in severe adverse events, and treatment-related fatalities were absent. Additionally, no additional severe adverse events were reported in the TBP group.

## Discussion

Immunotherapy, characterized by the use of immune checkpoint inhibitors (ICIs), has the potential to eradicate tumor cells by activating the anti-tumor immune function of patients’ own T lymphocytes. This therapeutic approach has gained widespread adoption within clinical settings (14). In cases of tumor progression among NSCLC patients undergoing PD-1 inhibitor immunotherapy, the optimal anti-tumor regimen and the feasibility of continuing PD-1 inhibitor maintenance therapy lack clear guidelines.

The concept of cross-line therapy, involving the replacement of chemotherapy drugs after first-line treatment progression, while retaining drugs that could provide ongoing benefit for second-line treatment, has been explored. These potentially beneficial drugs often complement cytotoxic agents, addressing aspects such as improved tumor vascularization or modulation of the immune microenvironment. Cross-line therapy has been investigated in diverse tumor types, including the combination of human epidermal growth factor receptor-2 monoclonal antibodies with chemotherapy in breast cancer treatment (15), and the application of anti-angiogenesis therapy alongside chemotherapy for colon and non-small cell lung cancer (16). These studies have imparted certain impacts on clinical practice.

Within the realm of lung cancer immunotherapy, a retrospective analysis of the OAK trial revealed that among 322 patients diagnosed with non-small cell lung cancer who received atezolizumab, disease progression occurred (9). Among these patients, those who persisted with atezolizumab treatment exhibited prolonged overall survival (OS) compared to those who received alternative treatments or no additional therapy. Similarly, the clinical investigation of nivolumab demonstrated that the TBP (treatment beyond progression) and NTBP (no treatment beyond progression) groups exhibited comparable overall survival (OS: 15.6 vs. 13.4 months; P=0.40). An analysis of real-world data from 134 instances indicated that the continuation of immunotherapy after progression can lead to extended survival, with a statistically significant benefit (OS: 17.2 vs. 7.5 months; p<0.01) (17).

The KEYNOTE-407 trial, evaluating the efficacy of PD-1 monoclonal antibody combined with chemotherapy versus placebo in the management of advanced lung squamous cell carcinoma, revealed that the group receiving immune combined chemotherapy had a mOS of 17.2 months, compared to a mOS of 11.6 months in the group receiving placebo combined chemotherapy (HR=0.71, 95%CI: 0.59-0.85) (18, 19). Our study included 41 patients diagnosed with advanced lung squamous cell carcinoma, among whom 28 received PD-1 monoclonal antibody treatment, resulting in a significantly longer median overall survival of 14.3 months compared to 6.0 months (p=0.003). This study underscores the potential benefits of continuous PD-1 monoclonal antibody administration for patients with lung squamous cell carcinoma.

While the IMPower133 (20) and KEYNOTE604 (21) studies have established the importance of immunotherapy in managing small cell lung cancer, limited literature exists on the effectiveness of PD-1 monoclonal antibody treatment for advanced small cell lung cancer following PD-1 therapy progression. Our study included 22 patients diagnosed with small cell lung cancer, of whom 16 were administered PD-1 monoclonal antibody immunotherapy. Although a positive trend of efficacy was observed, the lack of statistical significance could be attributed to the constrained sample size. Subgroup analysis focusing on pathological characteristics of the total lung cancer population demonstrated advantages in terms of mPFS and mOS for the TBP group compared to the NTBP group, with significant findings in patients diagnosed with squamous cell carcinoma.

Concurrently, a forest plot analysis revealed that age, gender, smoking status, ECOG performance status, surgical intervention, radiotherapy, metastasis location, and therapy line variables all favored the TBP group. Notably, the overall TBP group exhibited a lower ECOG score compared to the NTBP group, indicating that patients with superior overall health status are more likely to persist with PD-1 monoclonal antibody treatment after progression.

Furthermore, among the 141 patients who experienced disease progression when undergoing immunotherapy in this study, the TBP group (99) was compared to the NTBP group (42). This comparison was made in relation to prior literature from 2018 (7 vs. 87) (9), 2019 (60 vs. 116) (10), and 2020 (67 vs. 67) (11). The growing preference for TBP treatment options appears to stem from an enhanced understanding among clinicians of the effectiveness and safety of PD-1 monoclonal antibody therapy. Concurrently, the progressive reduction in pharmaceutical costs contributes to the increased utilization of extended treatment by individuals.

Nevertheless, this study bears certain limitations, including its nature as a single-center retrospective investigation with a limited sample size. Variability in the utilization of different PD-1/PD-L1 monoclonal antibodies and potential confounding factors must also be acknowledged. The assessment criterion adopted, RECIST1.1, while straightforward and expedient, may not fully capture the complexities of real-world clinical settings, impeding its widespread implementation.

The findings of this study suggest that the administration of PD-1 monoclonal antibodies to patients with advanced lung cancer experiencing progression, as determined by RECIST1.1 criteria, could lead to improved survival outcomes upon continued usage. The interval between the initiation of PD-1 monoclonal antibody therapy and the initial progression of the disease does not impact the correlation between PFS and OS subsequent to cross-line treatment. While prolonged immunotherapy administration is often linked to an increased incidence of adverse effects, our study demonstrated that the persistence of PD-1 monoclonal antibody therapy post-progression did not result in a higher occurrence of grade 3-4 adverse outcomes, indicating its tolerability. Therefore, the application of PD-1 monoclonal antibodies after disease progression could offer enhanced survival benefits with minimal unfavorable events. Larger-scale medical investigations are warranted to further elucidate the insights gained from this study.

## Data availability statement

The raw data supporting the conclusions of this article will be made available by the authors, without undue reservation.

## Ethics statement

The studies involving humans were approved by Ethics Committee of Jinling Hospital. The studies were conducted in accordance with the local legislation and institutional requirements. The participants provided their written informed consent to participate in this study.

## Author contributions

CC: Conceptualization, Investigation, Writing – original draft. XX: Data curation, Methodology, Software, Writing – original draft. YC: Formal Analysis, Project administration, Validation, Writing – review & editing. HG: Methodology, Software, Writing – original draft. WZ: Project administration, Resources, Validation, Writing – review & editing. FZ: Data curation, Software, Writing – original draft. CZ: Conceptualization, Investigation, Writing – original draft. SH: Funding acquisition, Software, Validation, Visualization, Writing – original draft. XL: Supervision, Writing – original draft.
